# Supplementary material for: Quantitative estimates of average geomagnetic axial dipole dominance in deep geological time
Source: Nat Commun. 2020 Nov 30;11:6100. doi: 10.1038/s41467-020-19794-7 (PMC7704635; doi:10.1038/s41467-020-19794-7)
Supplement: Supplementary file 1 — Supplementary Information [file 41467_2020_19794_MOESM1_ESM.pdf]

Supplementary Information

Quantitative estimates of average geomagnetic axial dipole dominance in deep geological time

Andrew J. Biggin, Richard K. Bono, Domenico G. Meduri, Courtney J. Sprain, Christopher J. Davies, Richard Holme, Pavel V. Doubrovine

Contents

Supplementary Note 1: Theoretical approximation of empirically obtained power law

Supplementary Note 2: Process for generating rescaled models

Supplementary Figure 1

Supplementary Figure 2

Supplementary Figure 3

Supplementary Figure 4

Supplementary Figure 5

Supplementary Figure 6

Supplementary Table 1

Supplementary Data.xlsx (Separate file)

Supplementary Note 1: Theoretical approximation of empirically obtained power law

Here we derive a power law similar to that obtained from our numerical dynamo simulations and observational models using purely theoretical considerations alongside various simplifications and approximations. Specifically, we consider only the case where the non-axial-dipole field comprises only the two equatorial dipole terms; we therefore neglect all terms with degree > 1. We also assume that Δ_i, the angular distance of the *i*th VGP from the geographic pole, which we denote in units of radians, to only ever be small. We stress that this power law was derived only to check the robustness of that described in the main text obtained using a much more rigorous approach.

At any one time instance, *i*:

$$\left(\frac{AD}{NAD}\right)_i = \left(\frac{{g_1^0}^2}{{g_1^1}^2 + {h_1^1}^2}\right)_i = cot^2\Delta_i \approx \frac{1}{{\Delta_i}^2} \qquad \text{S1}$$

Since VGP dispersion, *S* is latitude-independent in this scenario, it is equivalent to the Model G parameter a_r, defining S_r at the equator (here subscript *r* denotes the units of radians) :

$${S_r}^2 = {a_r}^2 = \frac{1}{N}\sum {\Delta_i}^2 \qquad \text{S2}$$

An estimate of the degree of axial dipole dominance, AD/NAD_{char}, can then be obtained combining S1 and S2:

$$\left(\frac{AD}{NAD}\right)_{char} \approx \frac{1}{{a_r}^2} \qquad \text{S3}$$

Taking logs:

$$log\left(\frac{AD}{NAD}\right)_{char} \approx -2\log a_r = 2\log\left(\frac{180}{\pi}\right) - 2\log a \qquad \text{S4}$$

Where *a* is the Model G parameter *a* defined in units of degrees

We now have AD/NAD and a_d in a power law form similar to equation (5) in the main text:

$$log\left(\frac{AD}{NAD}\right)_{char} \approx {k_1}^* \log a + {k_2}^* \qquad \text{S5}$$

Furthermore, the derived values of k_1^* (-2) and k_2^* (3.52) are reasonably similar to their empirically-obtained counterparts k_1 (-2.26) and k_2 (3.44). This degree of correspondence is somewhat reassuring as to the robustness of this power law. It may also be considered somewhat unexpected given that AD/NAD_{char} is not the same as AD/NAD_{median} (although the two are expected to be similar in value) and that we have neglected the entire nondipole field in this derivation.

Supplementary Note 2: Process for generating rescaled models

In Figure 3a and Supplementary Fig 4, the g_1^0 gauss coefficient (axial dipole) from three dynamo models and the field model GGF100k were rescaled in order to provide a further test of the robustness of the relationship between Model G a values and corresponding AD/NAD_{median} values.

The process for generating each point on Figure 3a (and curve on Supplementary Fig 4) was as follows:

1. For each of the four models, calculate AD/NAD_{median} prior to any rescaling and then iterate steps 2 and 3 below using rescaled values (denoted AD/NAD_{median}*) from the set {1, 2, 5, 10, 20, 50, 100}

2. To obtain each value of AD/NAD_{median}*, multiply g10 coefficients at all timesteps by a correction factor, *c* using:

$$c = \sqrt{(AD/NAD_{median} \;/\; AD/NAD_{median}{}^{*})} \qquad \text{S6}$$

   This provides a new time series of g10 coefficients (g10*)

3. Replace g10 terms in the original model's output with g10* keeping all other term identical such that the time series has the new ratio AD/NAD_{median}*. Apply the process outlined in *Methods* to obtain the best-fitting Model G *a* parameter using this new set.

In every case, modifying AD/NAD_{median} by an arbitrary amount simultaneously caused the Model G *a* parameter to shift in a manner consistent with the power law shown on Figure 2.

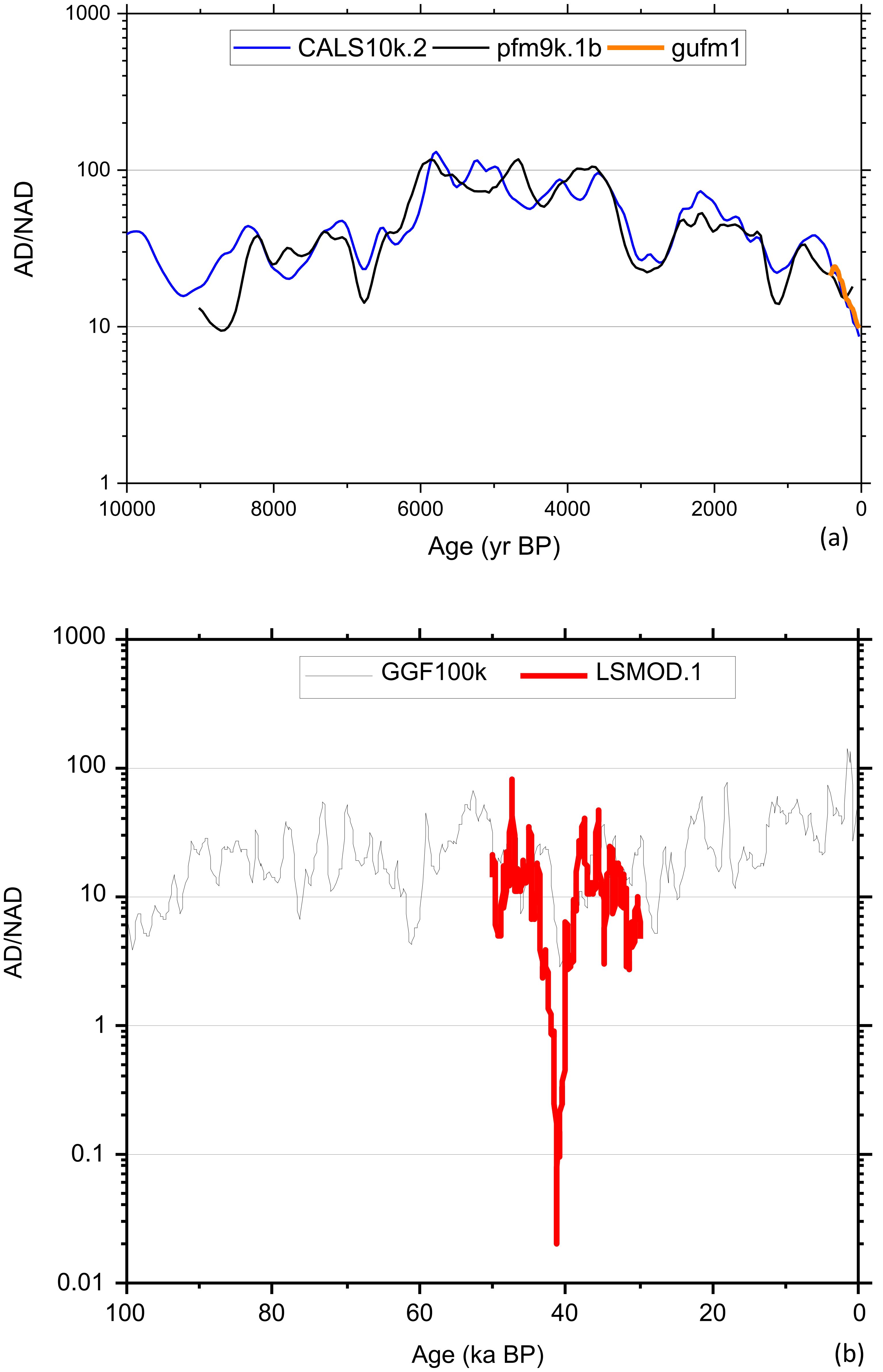

Supplementary Figure 1: Time series of AD/NAD at Earth's surface (note semi-log axes) for (a) gufm1[9]; pfm9k.1b[10]; CALS10k.2[8] and (b) LSMOD[7]; GGF100k[11].

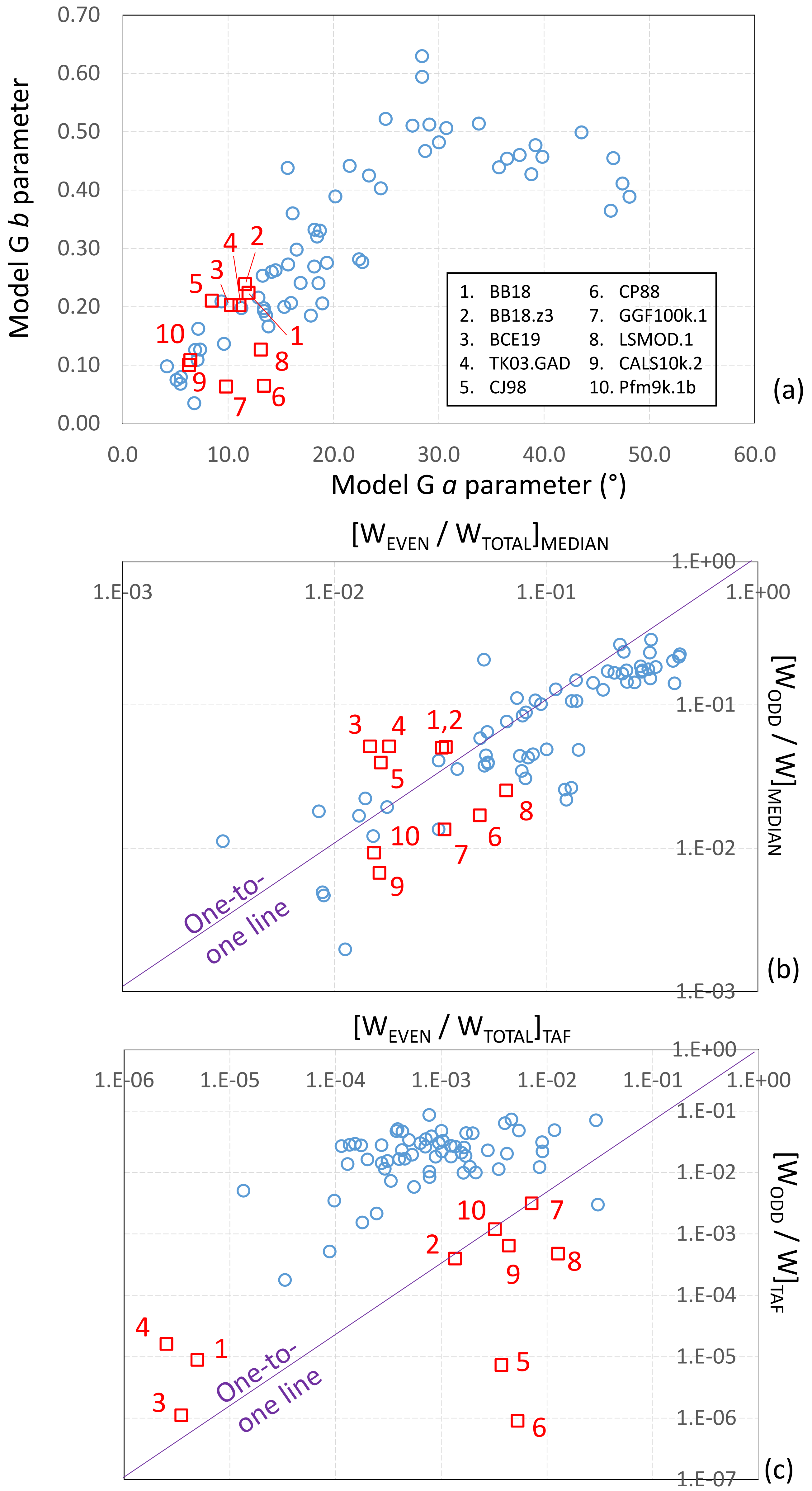

Supplementary Figure 2: Relationships between parameters describing surface field behaviour output from dynamo models (blue) and observational models (red squares). (a). Parameters of Model G –style fits to VGP dispersion results. (b,c) Relative Lowes power associated with Gauss coefficients whose degree and order sum to even and odd values (i.e. equatorially symmetric and antisymmetric terms respectively). In the odd case, the axial dipole is excluded. In (b) the Gauss coefficients are summed at each timestep and the median of the timestep values is used. In (c) a time-averaged field is first constructed by normalising polarity (all terms are flipped when axial dipole is reversed) and taking the mean of each Gauss coefficient; odd and even power sums are then calculated. There is clearly positive covariance in all three datasets. In (a), this indicates the tendency to be that, as equatorial VGP dispersion increases, so does the latitudinal dependence of the dispersion. In (b) scatter around the one-to-one line (purple) indicates that the non-axial dipole field at each time instance tends to be roughly shared between odd and even terms. In (c), the non-axial dipole part of the time-averaged field is shown to be between 1 and 2 orders of magnitude smaller than that, on average, at individual time instances but not equally partitioned into odd and even terms. Specifically, dynamo models tend to favour persistent odd terms whilst observational models tend to favour persistent even terms.

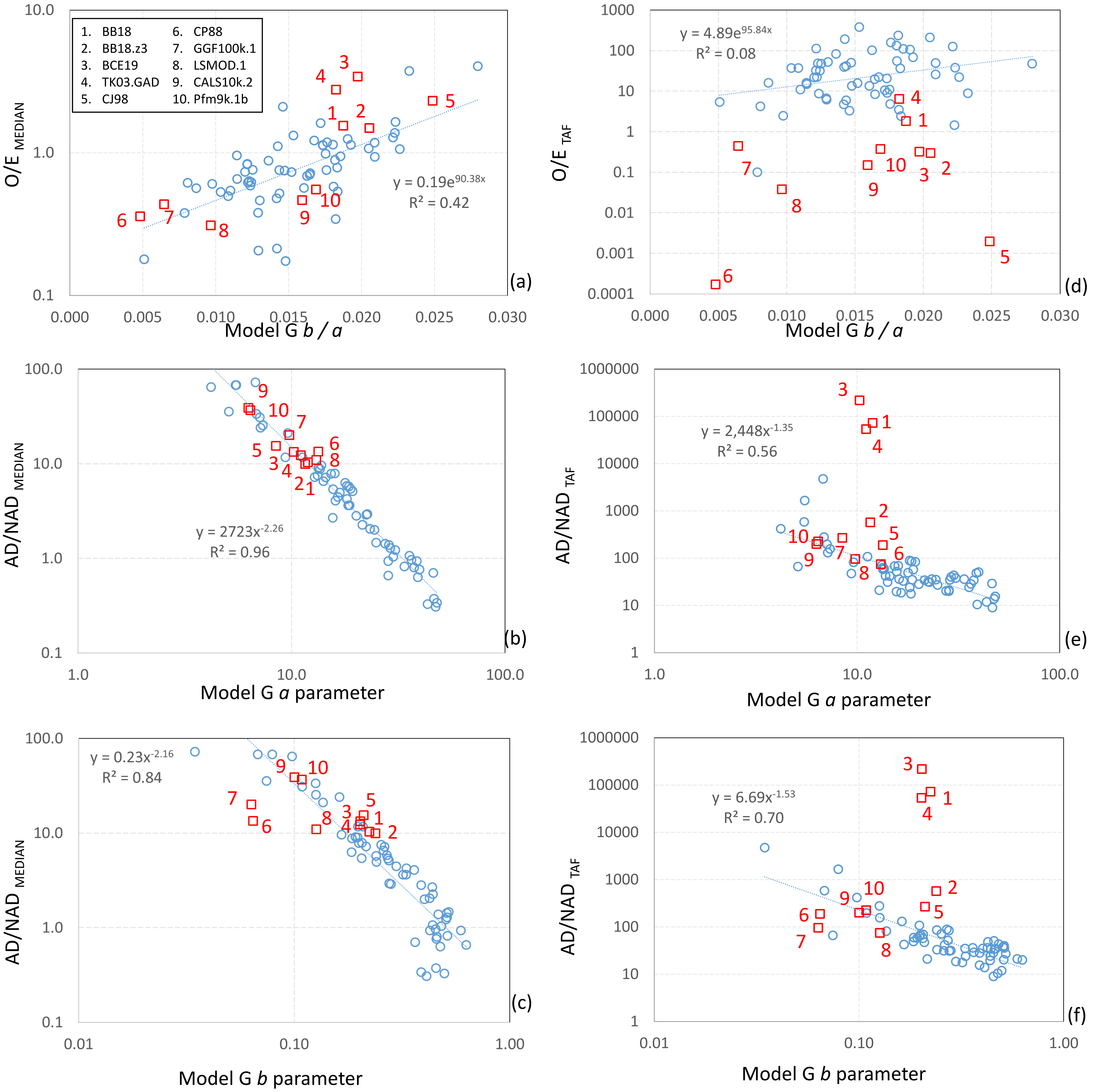

Supplementary Figure 3: Palaeosecular variation descriptors as predictors of surface field morphology described in terms of ratios of groups of Gauss coefficients for dynamo simulations (blue circles) and observational field models (red squares). In all cases, best-fitting lines and equations refer to the dynamo models only. (a, d) Ratio of Model G parameters shown versus ratio of Lowes power associated with groups of odd (excluding g_1^0) and even terms (i.e. equatorially antisymmetric and symmetricterms respectively). (b, e) Model G *a* parameter shown versus ratio of Lowes power associated with g_1^0 and all other terms. (c,f) Model G *b* parameter shown versus ratio of Lowes power associated with g_1^0 and all other terms. (a,b,c) are based on the median Lowes power ratio calculated at every timestep. (d,e,f) are based on the Lowes power ratio of the calculated time-average field (TAF).

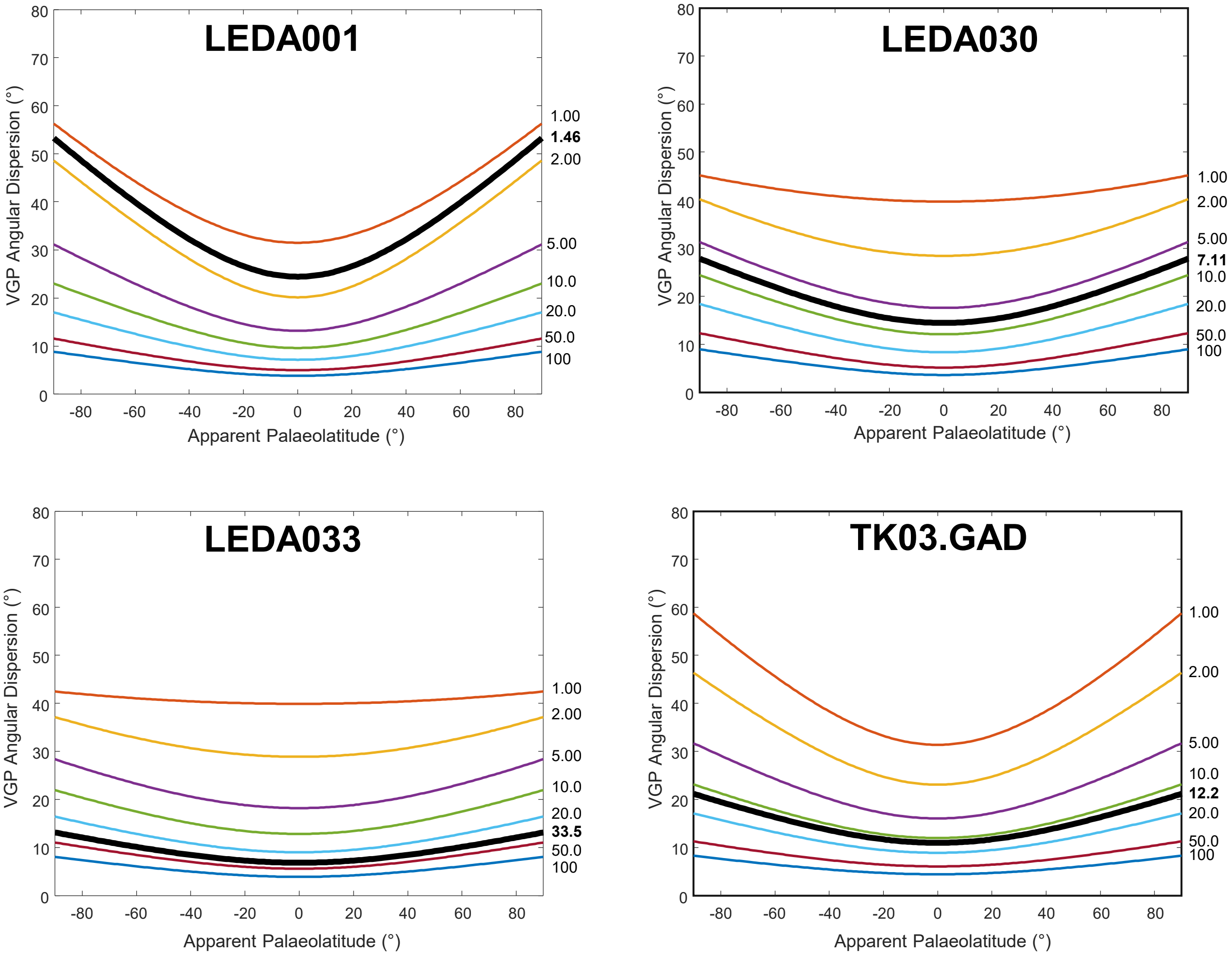

Supplementary Figure 4: Individual Virtual Geomagnetic Pole (VGP) dispersion vs Palaeolatitude plots for Model G datasets summarised in Figure 3a. Original fits are shown in bold and their axial dipole term is rescaled at each realisation to produce the AD/NAD_{median} values shown to the right of each plot. VGP dispersion values at the equator (defined by Model G *a* parameter in Figure 3a) are similar for all identical AD/NAD_{median} values regardless of the initial dominance of the axial dipole term.

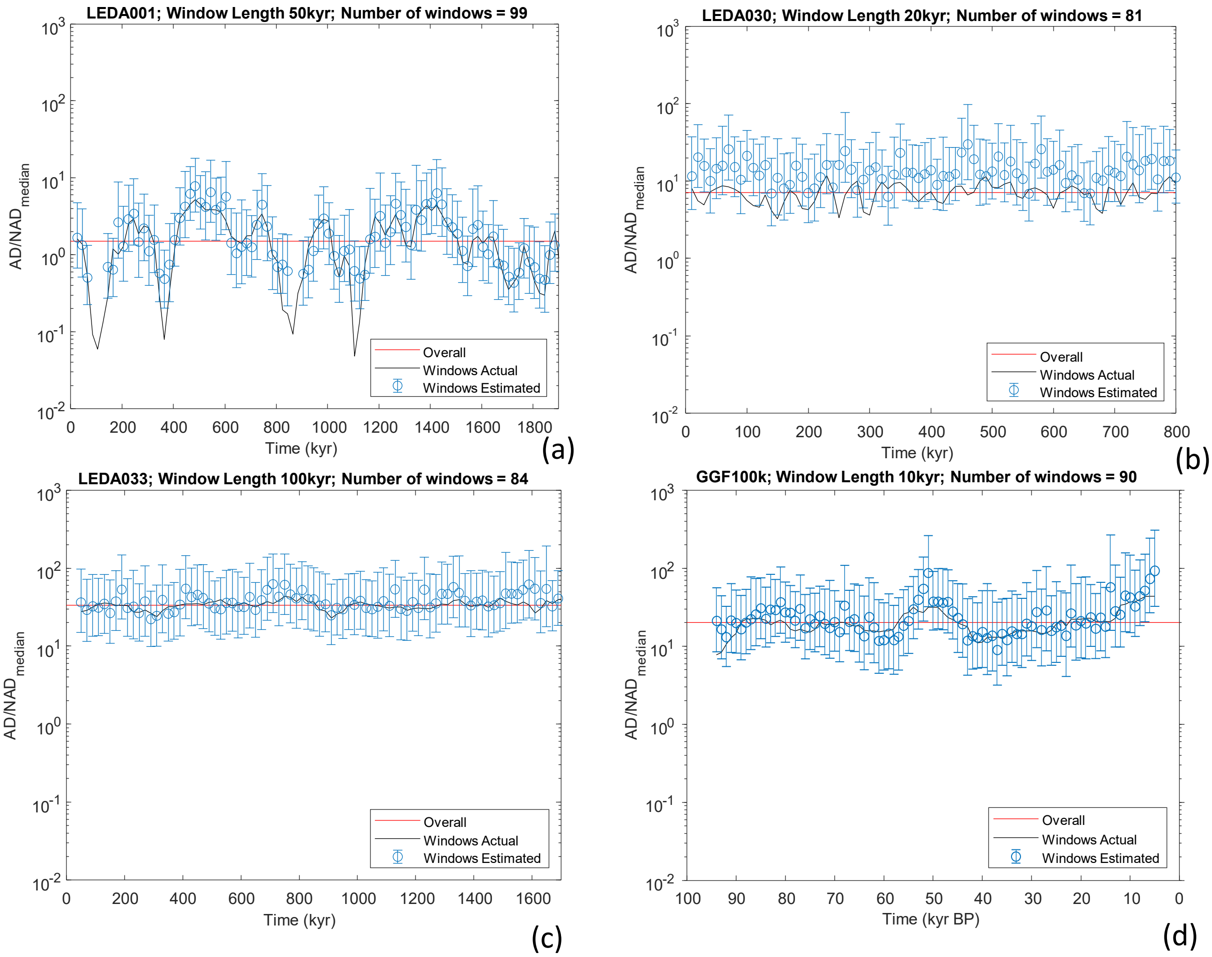

Supplementary Figure 5: Examples of four different time window lengths applied in a sliding window analysis to four different models. Smoothed values of actual AD/NAD_{median} are shown by a black line; individual estimates with uncertainties within windows are shown in blue; red lines show overall AD/NAD_{median} values for each entire model.

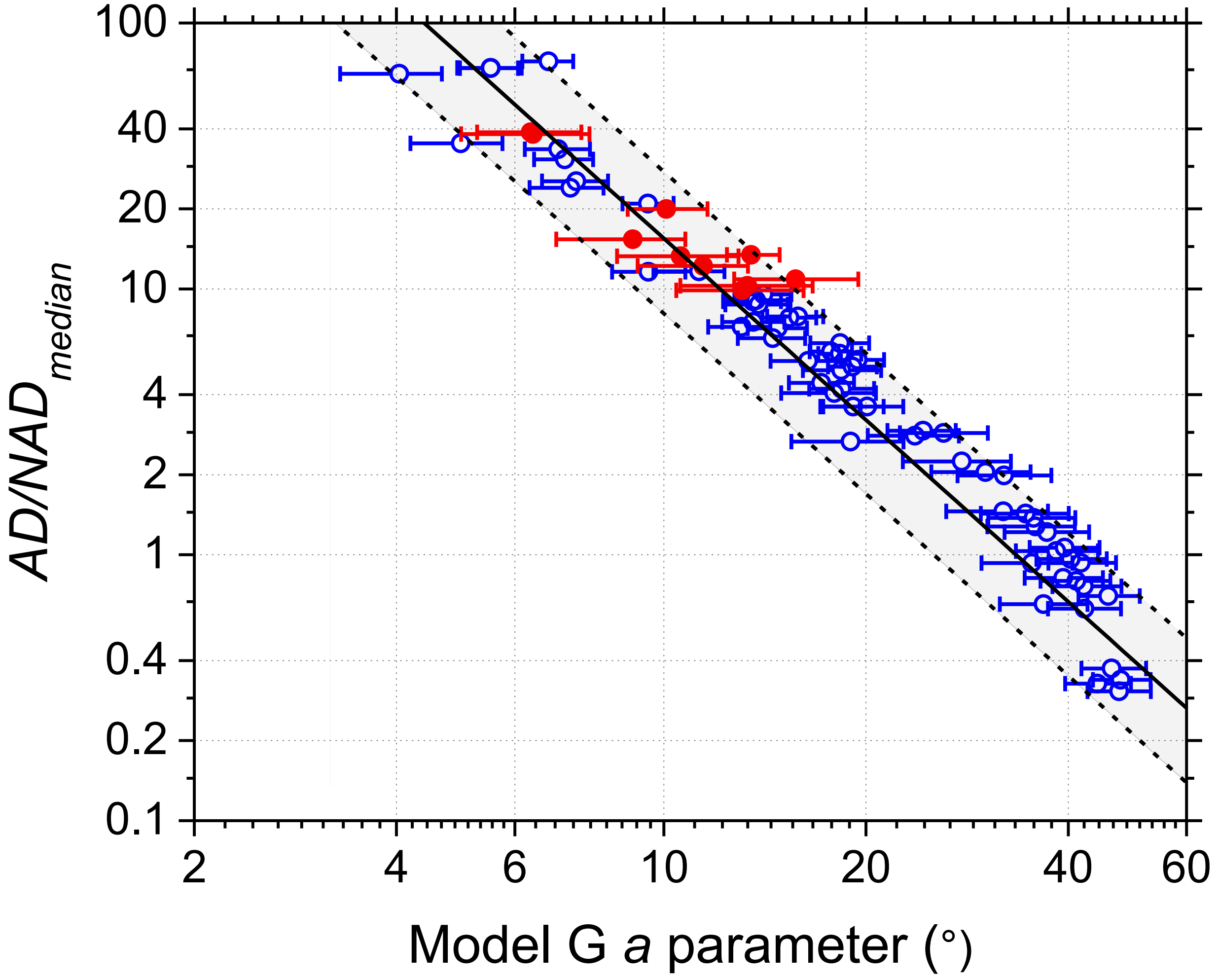

Supplementary Figure 6: An alternative test of downsampling to that presented in Figure 3c. Here, each model was down-sampled (again, 15 random timesteps at each of 19 random locations) 1000 times. The error bars represent 95% of the range of *Model G a parameter* values obtained from the 1000 iterations and circles are median values. Dashed lines are prediction bounds taken from Figure 2.

ID	Model	Ref	Duration (kyr)	Timestep (yr)	AD/NAD_{median}	O/E_{median}	AD/NAD_{TAF}	O/E_{TAF}	***a*** **(°)**	***b***	RMSE (°)
1	BB18	16	-	-	10.3	1.5	72462.6	1.8	11.9	0.22	0.99
2	BB18.z3	16	-	-	9.9	1.5	571.6	0.3	11.6	0.24	1.19
3	BCE19	17	-	-	13.3	3.4	218207.5	0.3	10.3	0.20	1.76
4	TK03.GAD	15	-	-	12.2	2.8	53551.9	6.4	11.1	0.20	1.30
5	CJ98	13	-	-	15.4	2.3	268.2	0.0	8.5	0.21	0.95
6	CP88	14	-	-	13.4	0.4	188.5	0.0	13.4	0.06	1.08
7	GGF100k.1	11	99.8	200	20.0	0.4	96.0	0.4	9.8	0.06	1.34
8	LSMOD.1	7	20.1	50	10.9	0.3	74.7	0.0	13.1	0.13	2.68
9	CALS10k.2	8	10	40	38.8	0.5	199.4	0.1	6.3	0.10	1.48
10	pfm9k.1b	10	8.9	50	36.6	0.6	225.5	0.4	6.4	0.11	1.83
11	gufm	9	0.4	2.5	16.4	0.4	21.2	0.4	6.0	0.00	2.97
12	IGRF	63	0.12	5	10.5	0.5	11.1	0.5	2.3	0.00	1.43

Supplementary Table 1: Summary properties of 12 published observation-based field models. In the case of giant Gaussian Process models (1-6), 10,000 realisations were used. AD/NAD_{median} is defined in the main text. AD/NAD_{TAF} is calculated using the same formula but using a single set of Gauss coefficients which are the arithmetic mean of those at each timestep. *O/E* is defined in ref-5 as the ratio of the sum of Lowes power (*W*)[4] in equatorially antisymmetric (odd) terms (after excluding g_1^0) to W in equatorially symmetric (even) terms. O/E_{median} is the median for all timesteps, O/E_{TAF} makes use of the time-averaged field as for AD/NAD_{TAF}. All are measured at Earth's surface. Parameters *a*, *b* and *RMSE* (root mean square error) refer to fits of Model G[14] to palaeosecular variation data extracted as set out in *Methods*. The lowest rows are shaded grey because the duration of these models are so short that the Model G parameters are almost certainly suppressed; they are therefore not included in any analyses.
