# Supplementary material for: Quantitative estimates of average geomagnetic axial dipole dominance in deep geological time
Source: Nat Commun. 2020 Nov 30;11:6100. doi: 10.1038/s41467-020-19794-7 (PMC7704635; doi:10.1038/s41467-020-19794-7)
Supplement: Supplementary file 3 — Description of Additional Supplementary Files [file 41467_2020_19794_MOESM3_ESM.pdf]

Description of Additional Supplementary Files

File Name: Supplementary Data 1

Description: Input parameters and summary properties of magnetic fields produced at Earth's surface by the numerical geodynamo simulations used in this study. 37 of these simulations appeared in previous studies (see references associated to the model names; names in parentheses refer to the nomenclature employed in ref-55). The input parameters, defined in Methods, are: the shell aspect ratio (ar), the Ekman number (E), the Rayleigh number (Ra), and the magnetic Prandtl number (Pm). All simulations have a Prandtl number Pr=1. Column 6 lists the temperature boundary conditions (BCs) employed: fixed temperature (FT) or fixed heat flux (FF), with the first two (second two) letters referring to the inner (outer) boundary. Columns 7 defines the type of heat flux lateral variations at the Prof Andrew John Biggin BSc PhD FRAS Chair of Palaeomagnetism Geomagnetism Laboratory Oliver Lodge Laboratories School of Environmental Sciences University of Liverpool Oxford Street Liverpool L69 7ZE UK T +44 (0)151 794 3460 F +44 (0)151 794 3464 E biggin@liv.ac.uk www.liv.ac.uk/info/staff/A208590 outer boundary (H for no lateral variations (homogeneous), r-Y20 for a recumbent Y20 pattern, T for variations after the tomographic model of ref-59). Columns 8 and 9 list ε, the amplitude of the heat flux lateral variations, and γ, the heat source/sink term amplitude, respectively (see Methods for more details). Columns 10 details the magnetic Reynolds number $Rm=Ud/\eta$, where U is the rms flow velocity. Columns 11 indicates the dynamo regime (D for stable dipole dominated solutions, R for Earth-like reversing solutions, and M for multipolar solutions). The regime is defined using the relative transitional time τT (column 12) which is the fraction of the total simulation time the true dipole pole latitude is further away than 45 degrees from the closest geographic pole. Following Sprain et al.49, we define D when $\tau T < 0.04$, R when $0.04 \leq \tau T \leq 0.15$, and M when $\tau T > 0.15$. Durations and timesteps were scaled assuming an outer core magnetic diffusion time of 200 kyr. Output parameters, AD/NADmedian is defined in the main text; AD/NADTAF is calculated using the same formula but using a single set of Gauss coefficients which are the arithmetic mean of those at each timestep. O/E is defined in ref-4 as the ratio of the sum of Lowes power(W)4 in equatorially antisymmetric (odd) terms (excluding g10) to W in equatorially symmetric (even) terms. O/Emedian is the median for all timesteps, O/ETAF makes use of the time-averaged field as for AD/NADTAF. Parameters a, b and RMSE refer to parameters of Model G18 fit to palaeosecular variation data extracted (see Methods). We note here that LEDA021 (Model 8) has a wrong Rm listed in ref-55. LEDA060 (Model 52) is incorrectly classified as a multipolar model in ref-49 due to a long initial transient that we excluded here. Values of Rm and τT generally do not exactly coincide with those reported in ref55 since we exclude the initial transients differently here.
